# Moving on: A survey of Canadian nurses’ self-reported transition practices for young people with chronic pain

**DOI:** 10.1080/24740527.2018.1484663

**Published:** 2018-06-22

**Authors:** Andrea Higginson, Paula Forgeron, Bruce Dick, Denise Harrison

**Affiliations:** a School of Nursing, University of Ottawa, Ottawa, Ontario, Canada; b Faculty of Health Sciences, University of Ottawa, Ottawa, Ontario, Canada; c Faculty of Health Sciences, School of Nursing, Dalhousie University, Halifax, Canada; d Departments of Anesthesiology and Pain Medicine, Psychiatry & Pediatrics, Faculties of Medicine and Dentistry & Rehabilitation Medicine, University of Alberta, Edmonton, Canada; e Faculties of Medicine and Dentistry & Rehabilitation Medicine, University of Alberta, Edmonton, Canada; f Nursing Care of Children, Youth and Families, Children’s Hospital of Eastern Ontario (CHEO), Ottawa, Ontario, Canada

**Keywords:** transition, chronic pain, young adults, adolescents, pediatrics

## Abstract

**Background:**

Practices to support the transition of a young person from the pediatric to the adult health care setting have been examined for many chronic illness populations. However, specific transition practices to support young people with chronic pain have not been examined.

**Aim:**

The aim of this study was to describe the current nursing practices used in the pediatric and the adult health care to support transition of young people with chronic pain in Canada.

**Methods:**

An online survey of pediatric and adult chronic pain nurses’ self-reported transition practices was conducted.

**Results:**

Twenty-two nurses completed the survey, 10 (45.5%) from the pediatric chronic pain setting and 12 (54.4%) from the adult chronic pain setting. Of the pediatric nurses surveyed none reported using a psychometrically valid tool to assess a young person’s readiness of general transition skills; however, one reported using a tool to assess understanding of chronic pain. Most health care facilities in which these pediatric nurses worked offered a general transition clinic, but only one of these facilities also had a chronic pain transition clinic. Nurses in both settings perceived that young people experience increased levels of distress during transition yet most did not report using formal transition practices in their care.

**Conclusion:**

Nursing practices and clinic resources to support the transition of young people with chronic pain may not meet the needs of this population. Practices may benefit from the use of psychometrically validated tools to assess general transition preparedness. Research is needed to adapt tools and determine best transition practices for the chronic pain population.

## Abstract (es)

RÉSUMÉ

**Contexte**: Les pratiques pour soutenir la transition d’un jeune d’un établissement de santé pédiatrique vers un établissement pour adultes ont été étudiées pour de nombreuses populations souffrant de maladies chroniques. Toutefois, les pratiques de transition visant particulièrement à soutenir les jeunes souffrant de douleur chronique n’ont pas été étudiées.

**Objectif**: Décrire les pratiques infirmières actuellement utilisées en matière de soins de santé pédiatriques et de soins de santé pour adultes afin de soutenir la transition des jeunes souffrant de douleur chronique au Canada.

**Méthodes**: Un sondage en ligne portant sur les pratiques de transition auto-déclarées par des infirmières pédiatriques spécialisées en douleur chronique et par des infirmières spécialisées en douleur chronique chez les adultes a été mené.

**Résultats**: Vingt-deux infirmières ont répondu au sondage; 10 (45,5 %) provenaient du milieu de la douleur chronique pédiatrique et 12 (54,4 %) provenaient du milieu de la douleur chronique chez les adultes. Aucune des infimières pédiatriques ayant répondu au sondage n’a déclaré utiliser un outil psychométriquement valide pour évaluer le degré de préparation à la transition d’un jeune; toutefois, l’une d’entre elles a déclaré utiliser un outil pour évaluer la compréhension de la douleur chronique. La plupart des établissements de santé dans lesquelles ces infirmières pédiatriques travaillaient offraient une clinique de transition générale mais seulement l’un de ces établissements avait aussi une clinique de transition en matière de douleur chronique. Les infirmières des deux milieux percevaient que les jeunes avaient des niveaux plus élevés de détresse pendant la transition mais la plupart ont déclaré ne pas avoir recours à des pratiques de transition officielles au moment de prodiguer les soins.

**Conclusion**: Les pratiques infirmières et les ressources cliniques pour soutenir la transition des jeunes souffrant de douleur chronique pourraient ne pas répondre aux besoins de cette population. Ces pratiques pourraient bénéficier de l’utilisation d’outils validés psychométriquement pour évaluer le degré de préparation générale à la transition. Davantage de recherche est nécessaire pour adapter les outils et déterminer les meilleures pratiques de transition pour la population souffrant de douleur chronique.

## Introduction

One in five children and adolescents in Canada experiences chronic pain,^1^^–4^ and a significant portion will continue to experience chronic pain into adulthood.^5,6^ Thus, a proportion of this population continues to require treatment for their chronic pain once they have reached the age of transfer to adult services. Determining the prevalence of these required services is undetermined because research on transition of the young adult with chronic pain into the adult health care system has not been a primary focus of research. Yet, without a coordinated and informed transition, young people with chronic illness are at risk for negative psychological and physical consequences^7^ and loss to follow-up, which can result in unnecessary crisis presentations to hospital emergency departments.^8–10^ Although there is a growing body of literature on the components, processes, and outcomes of transition for young people (adolescents and young adults) with other chronic illnesses,^7,11–13^ all findings may not be transferable to the pediatric chronic pain population due to the unique challenges of chronic pain (e.g., stigma and disbelief of chronic pain, individuality of pain and pain treatment, lack of formal chronic pain education for non-pain clinicians). Additionally, individuals with chronic pain report a lower quality of life than those with most other chronic illnesses,^14^ which could be further reduced by uncoordinated transition practices. Therefore, determining effective transition strategies to use with young people who have chronic pain could mitigate potential negative transition outcomes.

In 2016, the Canadian Association of Paediatric Health Centres (CAPHC) published guidelines to support the transition of the pediatric patient to adult care.^15^ These guidelines include three main components: person- or patient-centered care, a clinical approach, and system-level recommendations. The aim of the guidelines were to influence transition at the person and clinical levels, affecting change over time on a systems level. Most important, the guidelines identify the need for collaboration between all stakeholders and promote the use of tools and resources for transition as strategies to promote the successful transition of the pediatric patients with chronic illness. Although these guidelines are not specific to those with chronic pain and thus may not meet all of their transition needs, they do provide a foundation on which to build.

There are at least three main major factors that may act as barriers or facilitators to a successful transition for young people with chronic illness. They include the importance of independence to the young person, the shift in parental role, and the difference between health care systems.^16–19^ According to CAPHC, a one-size-fits-all transition is not possible.^15^ Transition supports need to be individualized, coordinated, and a collaborative process between the young person, family, and pediatric and adult care providers occurring over time and spanning both adolescence and early adulthood.^20^ The guidelines are meant to influence change and provide recommendations on transition from a person-centered, clinical, and systems-level approach. Therefore, specific strategies and supports for health care transition of young people with chronic pain are not identified.

Although health care transition requires support from all clinicians who work with young people and their families, nurses in particular can play a pivotal role in facilitating a positive transition experiences. Nurses are key clinicians on the interprofessional team who collaborate with others to assess and partner with patients and families to manage the effects of chronic pain and nurses coordinate much of a young person’s chronic pain care.

Psychometrically validated clinical tools have been developed to help determine transition preparedness for young people with chronic illness. These tools advance the measurement of transition readiness and are essential to better understand the interventions that are necessary to enhance the likelihood of engagement in the adult health care system.^21^ However, tools specific for the chronic pain population have not been developed and therefore it is unclear whether the tools that exist fully assess all of the factors that may present challenges to a young person with chronic pain as he or she approaches transition. Individual patient transition-related factors that require assessment are the age, disease knowledge, self-efficacy, executive functioning, and autonomy.^7^ Parents or family members also need to be prepared for transition by focusing on their ability to support their child’s growing capacity to manage their chronic pain and navigate the adult health care setting. Because parents and guardians have been the primary care providers, a shift in health management responsibilities must take place for a successful transition. Parents should also be supported to focus on strengths and abilities of the adolescent/emerging adult to develop positive, realistic, and developmentally appropriate expectations of the transition.^22^ Although there are similar transition readiness factors for all adolescents and young adults who face transition, those with chronic pain may require additional assessment, because the factors that have a negative impact on developmental trajectories of young people with chronic pediatric pain may be different. For example, school and social functioning challenges are unique (e.g., loss of friends since the onset of pain, decrease in school performance after the onset of pain)^23–25^ Futhermore, the management of allied health services (e.g., psychologist, physiotherapist service) posttransition may pose unique challenges in their access as they are a fundamental part of ongoing care.

Despite the recognized need to have a planned process for transition,^26^ there is little guidance on specific best practices for nurses working with young people with chronic pain and their families to support a successful transition. Moreover, guidelines geared toward nurses who work with recently transitioned young people with any form of chronic illness in the adult settings are lacking. Therefore, the purpose of this study was to gain an understanding of the current nursing care practices and clinic supports available in the pediatric setting pretransition and in the adult setting posttransition for young people with chronic pain and their families in Canada. Through the knowledge gained from this study, gaps in care will be identified and help design strategies to improve the health care transition specific to this population.

## Method

This study was a cross-sectional descriptive online survey designed to capture Canadian nurses’ transition care practices for adolescents and young adults with chronic pain and their parents along with their workplace system resources to support their practice. The survey was hosted on the Research Electronic Data Capture platform. Access to this survey platform was provided by the local university-affiliated children’s hospital. Given that nurses are comfortable with both online and pen-and-paper surveys,^27^ an online survey method was chosen due to the geographical distances between the researcher and potential participants. Additionally, in comparison to paper surveys, online surveys allow for shorter response times, cost effectiveness, and adaptability in question design.^28^ The shorter response times were due to the fast accessibility to the survey, the use of branching logic and adaptable questions, and immediate submission time. This is a particularly important factor in recruiting busy clinicians.

### Participants

A convenience sample of nurses who work in chronic pain clinics across Canada was recruited to participate in the survey. Nurses were the focus of the study because they are in a position to answer questions regarding health system supports in their setting that aid or hinder successful transition. Participants were recruited using several advertising approaches. One approach was posting the study on the Yahoo! Groups Canadian Pain Society Nursing Issues Special Interest Group listserve. A listserv is an email list manager, which is an opt-in email list and includes emails newsletters, announcements, and discussion groups within the email community.^29^ This particular listserv is a closed email list and includes nurses who work in the both pediatric and adult acute or chronic pain management across Canada. In conjunction with posting the online survey on this listserv, public posters were posted on faculty websites, as well as through social media. The survey was posted in major cities across Canada via an online classified advertisements website and the posting was also sent to clinically practicing chronic pain nurses personally known to the authors as well as promoted at a meeting of the Canadian Pain Societies Nursing Special Interest Group. Participants were invited from both the private and public chronic pain clinics, because young people may transition to either of these settings to access chronic pain care. The inclusion criteria for the study were (1) participants must be a registered nurse (RN) or nurse practitioner (NP) providing care in a chronic pain setting in either an adult or a pediatric setting and (2) could read and write in English. Exclusion criteria were (1) RNs or NPs who worked solely within the acute pain setting and (2) RNs or NPs who worked outside of Canada.

### Measures

There are no standardized measures to capture nurses’ transition care practices in the chronic pain setting. Therefore, a study-specific questionnaire was created based on the transition literature and published guidelines on best health care transition processes and informed by the nursing model on transition developed by Schumacher and Meleis.^30^ Based on this mid-range nursing theory, adolescents and young adults with chronic illnesses (such as chronic pain) experience two different types of transition when they are leaving the pediatric health care setting: developmental and situational.^30^

Developmental transitions are described as moving from one stage of life to another, such as infancy to toddlerhood or adolescence to adulthood. Adolescence is characterized by increased self-identity and independence. This developmental transition is also mirrored in the changes and responses in their parents’ role.^31^ Questions on the survey to capture these developmental differences included such questions as, “Do you assess the adolescent for their ability to self-manage their chronic pain without their parents’ assistance?”

Situational transitions are described as changes to roles and relationships that occur due to relocation,^31^ such as the changes to roles and relationships experienced by an adolescent or young adult with chronic pain and their health care provider as a result of the change in health care delivery services when they leave the pediatric health care setting and enter the adult health care setting health care. Despite the diversity in these transitions, inherent in both transitions is role acquisition, role loss, or the simultaneous loss of one role and gain of another.^31^ Questions on the survey to capture these situational differences targeted differences in the clinician roles. For example, we asked the adult nurses whether they provide support in advocating for school or university accommodations for younger patients, because this could be different compared to what the pediatric nurses provide.

The online survey allowed for the use of branching logic, which is when the response to a question will influence what questions will follow; thus nurses, who worked in the pediatric setting were only presented with pediatric health care specific questions. The same process was used for adult nurses, ensuring that adult nurses were only presented with adult health care specific questions. The questions in both the adult and pediatric versions of the survey were pilot tested by the co-authors and two experienced and practicing nurses in the field of chronic pain (one adult RN and one pediatric RN). Minor modifications to wording were made as a result of the pilot testing.

### Demographics

Three questions were asked to all participants to confirm eligibility prior to gaining access to the survey (participant was an RN or NP practicing in the chronic pain setting, practicing in Canada). Participants had to click yes to these three questions to gain access to the information and consent form. All consenting participants were asked demographic questions including province of employment and practice setting (pediatric or adult health care). Once those questions were answered, branching logic embedded in the survey directed them to either the pediatric- or adult-focused survey.

### Pediatric setting

All participants who identified that they worked with pediatric chronic pain patients were asked to answer 15 questions, based on the branching logic, using a combination of drop-down menus and open-ended text. See Appendix A for pediatric nurse survey questions. These questions captured information about their transition practices such as the age at which they transfer young people to adult programs, whether they engaged in transition discussions with patients and parents, and whether patients attended appointments (or portions of appointments) on their own in preparation for transition. An additional question was asked to capture nurses’ perceptions of transition-related distress in young people with chronic pain.

### Adult health care setting

The questions for the nurses who work in the adult health care system followed a format similar to those for the pediatric nurses. There were 13 questions with branching logic to capture their care practices, system processes, and resources to support their transition practices. See Appendix B for adult nurse survey questions. The questions were designed to capture data on policies that governed access to the clinic’s care to help determine whether a formal collaborative approach to the transition of the pediatric patients was in place (e.g., minimum age of patients, existence of formal transition clinics, process for referrals from the pediatric setting). Further questions were designed to capture the nurses’ perceptions of the young person’s ability to navigate the adult health care setting and participate in self-management. At the end of the survey, participants were asked an open-ended question to provide further information regarding their beliefs related to transition issues for adolescents and young adults with chronic pain seeking care in the adult health care system.

## Ethics

Ethics approval was obtained from University of Ottawa Research Ethics Board. This was an anonymous survey; links to users identification (e.g., Internet protocol—addresses, names, or initials of users) were not captured.

## Data analysis

A descriptive synthesis of the data was conducted using both narrative verbatim descriptive answers (for open-ended questions) and descriptive statistics (e.g., means, frequencies) for closed-ended questions. Descriptive statistics are useful for addressing research questions in studies that are primarily descriptive.^32^ This analytical approach provided an understanding of the current transition environment in both the pediatric and adult health care settings. The results are presented below in two sections, one synthesizing the results from pediatric nurse participants and the other synthesizing the results from the adult nurse participants.

## Results

### Demographics

Twenty-two registered nurses who work in the chronic pain setting provided consent for the study. Ten (45.5%) participants worked in the pediatric setting and 12 (54.4%) reported working in the adult health care setting. However, not all participants answered all questions. Nine out of the ten pediatric nurses completed the entire survey and the tenth nurse only consented to the study but did not complete the survey. Twelve nurses providing care in the public adult health care setting completed the screening questions and provided consent, but only nine completed any part of the survey. No participants reported working in the privately funded sector. There are approximately 180 RNs or NPs across Canada registered to the listserv; however, it is difficult to know whether they work in the acute or chronic pain field or the pediatric or adult health care setting. The nurses who responded from each province are as follows: British Columbia (*n* = 1), Alberta (*n* = 3), Ontario (*n* = 12), Quebec (*n* = 1), and Nova Scotia (*n* = 4). This question was asked pre–branching logic questions to ensure that they practiced in Canada and therefore the distribution of setting by province is not possible.

### Pediatric setting

All participants (*n* = 9) responded that they have a discussion with the adolescents in their clinic about transition. However, the participants varied in their responses as to which areas of transition preparedness were assessed. These areas included the adolescent’s ability to self-manage, understanding of his or her disease, understanding medication, and his or her ability to advocate for him- or herself. Furthermore, participants did not identify when these discussions began (just prior to transfer or a year or two prior to transfer). Seven of the nine nurses who responded indicated that they assessed adolescents for transition preparedness using informal assessment practices during a routine clinic visit. Nurses were least likely to assess an adolescent’s ability to navigate the health care environment (e.g., make their own appointments, have their medications filled), with only five of the nine respondents (55%) indicating they that they assessed this area of transition preparedness. None of these respondents used a formal set of questions to assess this area of assessment. All respondents indicated that they assessed adolescents’ understanding of chronic pain a part of transition preparedness and the majority (7/8; 87.5%) of nurses use a formal set of questions to conduct this assessment; however, no respondent identified using a psychometrically validated tool to conduct this assessment. See Table 1 for assessment of transition preparedness details.

**Table 1.** Assessment of transition preparedness in the pediatric setting.

	(*n*)	Yes (*n*)	No (*n*)	Formally assess (*n*)	Informally assess (*n*)	Transition tool (*n*)
Assessment of ability to self-manage their chronic pain (D)	9	7	2	1	7	0
Assessment of the ability to manage in the health care context (S)	9	5	4	0	3	0
Assessment of understanding of chronic pain (D)	8	8	0	7	7	1
Assessment of understanding of medication (S)	8	6	2	1	6	0
Assessment of understanding of psychological strategies to management pain (D)	8	8	0	2	7	0
Assessment of understanding of the physical approaches to pain management (D)	8	8	0	3	7	1
Assessment of ability to advocate for themselves in different settings (S)	8	7	1	1	6	0

D = developmental transition question; S = situational transition question; *n* = total number of participants who answered this question; Informally assessed = informal discussion on the topic during patient encounters; Formally assessed = planned sets of questions asked during planned patient encounters; Transition tools = use of standardized transition assessment tools.

Most of the participants (*n* = 6) identified that their health care facility has a general transition clinic that they could refer the adolescent to for transition assessment; however, only one participant indicated that they had a formal chronic pain transition clinic with adult health care providers. In terms of resources to help with transition (i.e., support groups, after-hours telephone support) for adolescents and their parent(s) or guardians, the majority of the participants (*n* = 8) indicated that those additional supports were not available. Moreover, the majority of participants (*n* = 6) identified that they did not provide literature to the patient and family on transition. In terms of collaboration between the two health care systems, the majority of the pediatric nurses (*n* = 6) indicated that their transition process consisted of sending a letter to the adult chronic pain physician from the pediatric physician and interdisciplinary team, as well as a follow-up phone call to the adult chronic pain team. In addition to the collaboration between pediatric and adult health care providers, few participants (*n* = 3) identified that they had a final face-to-face meeting with both teams (pediatric and adult healthcare providers) to complete the transition. Please see Table 2 for pediatric system supports.

**Table 2.** Pediatric transition system supports (*n* = 8).

	Yes (*n*)	No (*n*)
Does your health care facility have a transition clinic to refer the patient to?	6	2
Do you have a formal chronic pain transition clinic with adult health care providers?	1	7
Is there a formal support group available for adolescent seeking support with transition	3	5
Is there a support group available for the parents or guardians of the transitioning adolescent seeking support with transition?	0	8
Do you offer any patient literature on the transition experience?	2	6

### Adult health care setting

Most of the nurses (seven out of nine) who identified as working in the adult health care setting indicated that the chronic pain clinic where they worked accepted patients referred from pediatric chronic pain clinics. The other two adult nurse participants were unsure whether their patients were transitioned from a pediatric chronic pain clinic. Of the participants who reported caring for transitioned patients, few (*n* = 3) identified a formal collaboration between pediatric and adult health care providers on the multidisciplinary teams (physician, registered nurse, psychologist, and physiotherapist). One participant identified that they did not contact the pediatric clinicians, because the patient would arrive for his or her first appointment with a written copy of their medical history from the pediatric chronic pain physician. See Table 3 for the adult chronic pain nurses’ transition processes and procedures.

**Table 3.** Adult chronic pain nurses and transition process and procedures.

	(*n*)	Yes (*n*)	No (*n*)	Unsure (*n*)
1. Are you referred patients who were treated in the pediatric setting?	9	7	0	2
a. If yes, would your pain treatment facility contact the pain treatment facility of the transitioned young patient?	6	5	1	n/a
b. If yes, do you have a formal discussion with the referring:	3	Physician: 2; Registered nurse: 3 Psychologist: 2; Physiotherapist: 1		
c. If no, does the patient come to the first appoint with a written copy of their medical history?	1	1	0	n/a
2. Do you receive a referral letter from the services?	9	8	1	n/a

The majority of participants (*n* = 8) in the adult health care setting reported that they did not obtain formal consent from patients to allow parents or guardians to attend the appointments with adolescent or young adult patients; however, four out of the nine participants reported that they would be required to obtain a formal consent when it came to sharing information with the parent or guardian. The majority of respondents (*n* = 7) identified that they would include parent(s) or guardian(s) in the first appointment, and six out of the seven reported that parent(s) or guardian (s) were only included for part of the appointment. In terms of health care navigation, such as calling for appointments and refilling prescriptions, the majority (*n* = 6) reported that they would allow parent(s) or guardian(s) to perform these tasks; however, most (*n* = 6) noted that this would require formal consent. Please see Table 4 for parental involvement after transitioning to the adult health care system.

**Table 4.** Adult chronic pain nurses’ process for parent involvement.

	(*n*)	Yes (*n*)	No (*n*)
1. Do you obtain a formal consent from the patient to allow the parent/guardian to attend the appointments?	9	1	8
2. Does your facility obtain a formal consent from the patient to share health information with the parent or guardian?	9	4	5
3. Do you include parents or guardians in the first appointment?	9	7	2
a. If yes, do parents attend the entire or part of the appointment?	7	Part of the appointment: 6	Entire appointment: 1
4. Do you permit parents to attend further appointments?	9	7	2
5. Do you permit parents to call for appointments, refills, update information, pain management advice?	9	2 Only if consent was given: 6	1

In terms of transition preparation, most of the participants perceived that young people were not prepared for the adult health care setting, and none of the adult chronic pain nurse participants reported the ability to offer supports to the transitioning patients after clinical hours. The majority (*n* = 7) of the adult chronic pain nurses responded that support groups for patients with chronic pain were available at the clinic where they worked; however, the average age of the support group attendants was between 40 and50 years of age, and only two participants identified the availability of a support group for young adults. Only four participants responded that they had the ability to provide further supports in terms of assistance in securing school or work accommodations. These four participants supported the newly transferred patient in securing accommodations by providing a letter or telephone call to the appropriate institution (i.e., employer or university). Table 5 summarizes adult health care nurses’ perceptions of patients’ transition preparations and provided supports.

**Table 5.** Adult chronic pain nurses: Patients’ transition preparations and provided supports.

	(*n*)	Yes (*n*)	No (*n*)
1. Do you find that newly referred transitioned patients are prepared for treatment in the adult health care system?	9	2	7
2. Does your clinic provide support to newly transitioned young adult patients after hours	9	0	9
3. Does your clinic offer group sessions to people living with chronic pain	9	7	2
a. If yes, does your clinic offer sessions to young adults only?	7	2	5
4. Do you provide support in advocating for school or university accommodations for younger patients?	8	4	4

## Perceived distress

The majority of participants from both the pediatric and adult health care settings indicated that they believed that patients experience distress (e.g., anxious feelings) around the time of transition. All of the pediatric nurse participants believed that young people experienced an increase in distress prior to transition, whereas only four out of the nine adult nurse participants perceived that newly transitioned young people experienced distress related to being transferred to the adult health care setting. The other five adult nurses reported that they were unsure whether their newly transferred patients experienced distress as a result of leaving the pediatric health care setting to access care in the adult health care setting. A narrative verbatim descriptive summary of the one question that asked participants for their perceived reasons for why young people may experience distress as a result of transition is provided in Table 6.

**Table 6.** Participants’ perceptions of reasons for patients’ stress and anxiety due to transition.

Pediatric system *N* =8	Adult health care system *N* = 4
Multiple transitions! Not just from us, usually from their primary care providers, other subspecialties PLUS life transitions—school, work, moving away Fear of unknown. New team to get to know Uncertainty about their future pain management They are anxious about the unknown within the adult health care world. Adolescents are also anxious about advocating for themselves once they are transitioned Perceived minimal access Most adolescents are aware that (generally speaking) adult services involve a more self-management approach Due to the unknown and uncertainty. Unfamiliar with new medical teams and clinics. Leaving a hospital or clinic they have built relationships with	It is all new to them. They have been with professionals for usually many years and now we are completely changing that along with maybe how we deliver care in adult world Expectations, immaturity, and communications skills require development/coaching Unrealistic expectations of care in tertiary care setting Some, particularly those who have worked with pediatrics for years, have difficulty with working with a new team

## Discussion

This study explored the transition practices and perceptions of pediatric and adult nurses who are involved in the care of adolescents and young adults with chronic pain. Despite research indicating that successful transition is a process and that poorly planned and executed transition can lead to negative health outcomes (e.g., loss to follow-up, increased use of emergency departments for health management),^8–10^ formal transition processes for young people with chronic pain across Canada are early in their implementation. The guidelines from CAPHC were only recently released (in 2016) and therefore may not have been widely distributed; nevertheless, adolescents and young adults with chronic pain need to be prepared and supported during the transition process. The majority of the pediatric chronic pain nurses who participated in this study reported that they informally address transition with their patients, which is not surprising given that validation studies on transition tools are relatively new. However, informal transition, although well intended, may result in varying practice between and within clinics, meaning that not all young people with chronic pain may benefit from best practices. For example, without using a standardized tool to assess general transition readiness, gaps in the young person’s knowledge and skills may go unaddressed. Although standardized and tested transition readiness assessment tools designed specifically for young people with chronic pain are not available, two self-report tools (TRANSITION-Q and Am I ON TRAC) have been identified by CAPHC as appropriate for assessing health-related knowledge, self-efficacy, and skills for transition.^15,33,34^ TRANSITION-Q in particular is a unidimensional scale consisting of 14 questions capturing self-reported transition readiness behaviors of young people (e.g., I speak to the doctor instead of my parents), and scores discriminate between those who voice readiness to transition and younger patients.^33^ Moreover, it has been used with youth 12 years of age and older to help measure and track the development of generic transition skills over time.^33^ Tools such as TRANSITION-Q could be used in pediatric chronic pain clinics to help identify transitional knowledge and skill gaps in need of targeted interventions for individual patients. Despite not being specific to the chronic pain context, these tools do focus on knowledge and skills needed by all transitioning young people and could provide a foundation on which to build. The evidence suggests that transition planning must start as soon as possible (some stating by age 12 years) to help build knowledge, skills, advocacy, and self-efficacy in young people over time,^7,15,26^ and there is no indication at this time that this practice should be different for young people with chronic pain. Nevertheless, the development and testing of specific transition tools targeting the chronic pain population would be beneficial because they may have additional transition needs (e.g., how to manage stigma during transition, how to access non-physician care). Challenges in the clinical implications for assessing for transition preparedness may not be determined at this time because these assessments are not presently the standard of care; however, incorporating these clinical assessments could have significant benefits to the young person with chronic pain and their parents for preparing for the future.

Interestingly, the majority of the participants who work in the adult health care system perceived their young patients who were transferred from a pediatric health care setting as not adequately prepared. However, these nurses indicated that there were minimal resources to support young people after transitioning to the adult health care setting, perhaps suggesting that transition is viewed more as an event (transfer) within the adult health care setting rather than a process in which they continue to have a role. Only one pediatric nurse participant reported that an adult chronic pain clinician attended the last pediatric clinic appointment prior to transferring the young adult to the adult chronic pain clinic. Research suggests that effective supports offered to young people posttransition could include separate young adult care clinics and out-of-hours phone support^35^; however, the nurses in this study did not identify the availability of these interventions. Health care transition is a responsibility for both pediatric and adult clinicians and can only be achieved through the collaboration of providers representing both sides of the health care system,^36^ but this does not appear to be the practice within the Canadian chronic pain context at present.

Young people with chronic illnesses have reported experiencing distress (e.g., fear, anxiety) from the unknown when changing health care providers; however, transition preparation has been found to alleviate these feelings.^17^ Rutishauer and colleagues reported that the greatest barrier for young people during transition is the attachment and the importance of the relationship with the pediatric clinician,^35^ suggesting that pediatric clinicians need to help young people establish a trusting relationship with the adult health care clinicians. Entering in a new health care system and meeting a new clinician can invoke feelings of anxiety. Given that distress (e.g., anxiety) about the transition is the second greatest barrier to a successful transition, it is critical that strategies to manage transition distress (e.g., inviting young people to attend all or part of their clinic appointment without their parents in the pediatric and adult settings; meeting the adult health care providers prior to transition; and visiting the new health care facility) be instituted in both pediatric and adult health care settings.^35^ Increases in transition-related distress (fear, anxiety) may be especially salient for those with chronic pain because these types of psychosocial factors are associated with the prevalence of a variety of different chronic pain types,^3^ and anxiety in particular is associated with exacerbations of pain and pain-related disabilities.^37^ Although the pediatric nurses reported the existence of a nonspecific transition clinic in their hospital, they did not report that all of their patients were referred to this service or whether they were referred early enough to gain the needed knowledge and skills for transition. Research is needed to explore whether early assessment, preparation, and support decrease transition-related distress for young people with chronic pain. However, despite the need for more research specific to tool development for youth with chronic pain, based on current recommendations and findings, general transition care (e.g., using standardized tools, referring young people to nonspecific transition clinics with enough time to build skills) should be provided to support young people with chronic pain. Knowledge translation studies would be helpful to determine the barriers and strategies to implementing transition practices for youth with chronic pain.

Additionally, research suggests that parents require transition preparation to enable them to identify their child’s strengths and weaknesses to encourage them to develop positive and realistic expectations to improve their transition.^22^ Collaboration between health care providers and parents will provide parents with the essential transition information and resources to enable youth to engage in self-management, throughout the transition process, because parents contribute greatly to the development of independence.^38^ However, our findings note that at present, nurses may not be maximizing support for parents, because they may not be formally assessing parents’ abilities to support their child’s transition. Parental involvement with transition is an important component of a successful transition; therefore, the parents require assistance to support, encourage, and problem-solve transition-related challenges with their child.^17^ This finding may not be limited to nurses who work with patients with chronic pain and may extend to other clinicians of the team. Because parents experience increased levels of distress during the transition period,^16^ they may benefit from targeted interventions by nurses and or other interprofessional clinicians. This may be especially important for parents of young people with chronic pain due to pain-specific features (e.g., lack of understanding about chronic pain in society) and because many of these parents experience levels of clinically significant distress, including anxiety and depressive symptoms, and parental role stress.^24^ One strategy that may be helpful to parents is to welcome them into appointments in the adult health care setting, at least in the short term, because adolescents and young adults are not always able to voice important issues.^39^ Witnessing that their child is comfortable in discussing their needs with their new health care team may help alleviate some parental concerns, and if their child is not comfortable, parents’ will be in a better position to support their child by knowing the proposed treatment. Clearly, research is needed to determine the best ways to support parents of young people with chronic pain through this process, in both the pediatric and adult health care settings.

Transition resources presently available for this population may be limited. For example, the reported ages of the support group members in the adult chronic pain health care context in this study were substantially older than the ages of newly transitioned young people. Though this is largely a result of the epidemiology and age distribution of chronic pain in the general population, more attention to programming for young adults with chronic pain is important. Peer support from a person with a similar chronic illness has been described as helpful in managing life as an adult, and peers are helpful during the transition process because they provide opportunities to share similar experiences and coping strategies.^16^ Peers may also provide a feeling of belonging and understanding by providing a milieu to talk openly about one’s pain problem without the impression that these peers are disinterested.^40^ However, being confronted by older patients who have experienced a chronic illness for a longer period of time can result in young people feeling more fearful about their own future.^11^ In addition, it may be that the prognosis for young adults with chronic pain may be quite different from that of middle-aged or older adults with chronic pain and may give young adults an incorrect impression regarding their future potential. Therefore, adult chronic pain clinics should consider offering support groups that are mindful of the age differences and varying needs between their younger patients and older patients.

In this study, the majority of nurses working in the adult chronic pain clinics reported believing that young adults were not prepared to receive care in the adult health care system. Yet, most of these nurses did not report being participants in a transition process to support young people but rather received young people after being transferred (e.g., only receiving written discharge letters or a phone call about the young person); thus, it may not be surprising that young people are not prepared to navigate an unfamiliar health care setting. Despite the small sample size, it does suggests that coordination of a transition process for this complex and at-risk population is not in place but rather that young people are transferred to the adult health care setting as a one-time event. This is not surprising, because Suris and colleagues purport that the principles of a successful transition are not well applied within adult health care^41^ for any health condition. Furthermore, the adult health care system continues to be described by young adults as impersonal and disease focused and report that it is difficult to establish relationships with health care providers.^16^ These perceptions of the adult health care system could be changed through transition preparation. Moreover, Gorter and colleagues suggest that through the implementation of a collaborative written transition plan (including members such as pediatric and adult clinicians, educational institutions, community services), young people and their parents will be provided with the information they require to make informed decisions in the future.^43^ However, implementation of policies for clinical transition practices should be evaluated longitudinally to assess long-term outcomes.^42^ This supports the need for research to determine whether furthering the involvement of adult chronic pain providers in the transition process improves the experience and outcomes for young people with chronic pain.

Research suggests that an interprofessional approach is the most effective way to treat chronic pain.^14^ This approach is generally in place in the pediatric chronic pain clinics in Canada. Unfortunately, this is not necessarily the case in adult chronic pain clinics where case loads are significantly greater. To date, transition processes have had a medical focus with an emphasis on the role of the physician.^43^ However, given the significant role of other modalities in the management of chronic pain, research into the transition care practices of other professional groups (such as psychologists, social workers, physiotherapists, and occupational therapists) is warranted because young people with chronic pain rely on these other professionals to provide therapeutic interventions.

## Limitations

Despite the contribution this study makes to understanding of the transition practices for young people with chronic pain, there are several limitations. First, the sample size was small, particularly in terms of participants who worked in adult chronic pain clinics. There are multiple chronic pain clinics across Canada, both private and public. Adolescent and young adult patients may transition to either sector, and supports available in the private sector may differ from those available in the public health care system. However, no participants in this study identified as working in the private sector; therefore, information about the private sector was not captured. Moreover, despite multiple outreaches to recruit more nurses who work with chronic pain populations in adult health care, the sample size remained small, perhaps suggesting that adult nurses view transition as an event and not a process or perhaps that nurses are not knowledgeable on the concept of transition and thus not interested in participating. Second, the survey was limited because it addressed the perceptions and self-reported practices of nurses working in these systems; however, because many of the chronic pain clinics in Canada are interprofessional, other health care providers may carry out some of the transition care. Nevertheless, nurses generally coordinate care and thus it is unlikely that overall transition practices differ among members of the interdisciplinary team. Finally, the questions on the survey asked about transition as a process, but it is unclear whether those who completed the survey have the same understanding or whether they interpreted transition as the one-time event of transfer from the pediatric setting to the adult setting. This could possibly be due to the confusion because the survey did not differentiate between transfer (the action of receiving care by a different health care provider) and transition (the gradual preparation process to becoming prepared and receive care in the adult health care setting); however, this confusion was not mentioned in the development of the questionnaire during the pilot process. Future studies on transition should clarify and define these terms. .

## Conclusion

Transition to adulthood and the adult health care setting is inevitable for all adolescents with a chronic health condition, including those with chronic pain. Despite published guidelines for best practice in general transition care, there are no uniform transition processes and practices to support young people with chronic pain in Canada. Most nurses who work in pediatric chronic pain clinics assess transition readiness informally. The use of standardized general transition assessment tools could identify gaps in some aspects of transition readiness and identify areas requiring intervention. Transition supports and programming in the adult health care chronic pain setting are limited, and this may have a negative impact on health care engagement posttransition. Through collaboration, pediatric and adult health care providers could help create a more seamless transition, which may have a positive impact on the transition experience and overall health outcomes of young people with chronic pain in Canada.

## Appendix A: Pediatric setting survey

1. At what age do you transition adolescents to adult health care services?
2. Does the health care team discuss transition with the adolescent patient with chronic pain and their parents?
  - No
  - Yes. If so, when do you have this discussion?
    - Last visit
    - 6 months before transition
    - 1 year before transition
    - Over one year
3. Do adolescent patients attend the whole or part of appointments without their parent or guardian?
  - Yes
  - No
4. Do you assess the adolescent for their ability to self-manage their chronic pain without their parents’ assistance?
  - No
  - Yes. If so, how is this assessed in your setting? (Open box to type in additional response)
5. Do you assess the adolescent for their ability to manage the health care contexts (e.g., make appointments, call for prescription refill, advocate for themselves)?
  - No
  - Yes
6. Do you assess the adolescent for their ability to understand chronic pain?
  - No
  - Yes
7. Do you assess the adolescent’s understanding of their medications?
  - No
  - Yes
8. Do you assess the adolescent’s understanding of the psychological strategies to help manage pain?
  - No
  - Yes
9. Do you assess the adolescent’s understanding of the physical approaches to help manage pain?
  - No
  - Yes
10. Do you assess the adolescent’s or young person’s ability to advocate for themselves in different settings (i.e., with employers, teachers, professors) about their chronic pain condition?
  - No
  - Yes
11. Is there a support group available for the adolescent or young adults and their guardian or parent to seek support with transition?
  - No
  - Yes
12. Do you offer any patient literature on the transition experience?
  - No
  - Yes
13. Does your health care facility have a transition clinic to refer the patient to?
  - No
  - Yes
14. Do you have a formal chronic pain transition clinic with adult health care providers?
  - No
  - If no, how do you collaborate with adult health care workers
    1. Referral letter from MD
    2. Referral letter from each health care provider (e.g., psychologist, physiotherapist, physician, nurse)
    3. Discussion over the phone with the adult health care providers
    4. Meeting face to face with adult health care providers
  - Yes
  - If yes, can you briefly describe what the pediatric and adult pain services team do together? (open-ended question for free text)
  - Do all adolescents have the same formal transition process?
    1. No
    2. If no, why not (open text)
    3. Yes

15. Is there anything else we should know about your transition practice? (Open-ended question for free text)

## Appendix B: Adult health care setting survey

1. Is your clinic private or publicly funded?
  - Private
  - Public
2. Does the provincial government fund your pain clinic?
  - No
  - Yes
3. Are you referred patients that were treated in the pediatric setting?
  - No. **If no, then survey is completed at this point.**
  - Yes
4. Does your pain treatment facility ever contact the pain treatment facility of the transitioned young patient?
  - No
  - If no, does the patient come to the first appoint with a written copy of their medical history?
    - No
    - Yes
  - Yes
  - If yes, do you have a formal transition clinic with the pediatric center?
    - No
    - Yes
5. Do you have formal discussions with the referring: (tick box for all that apply)
  - Physician
  - Registered nurse
  - Psychologist
  - Physiotherapist
  - Other:
6. Do you receive a referral letter from the services **only**?
  - No
  - Yes
7. Do you include the parents in the appointment?
  - No
  - Yes
  - If yes, do parents attend the entire or part of the appointment?
    - Part
    - Entire
8. Do you permit parents to attend further appointments?
  - No
  - Yes
  - If yes, for how long do you permit parents to attend appointments with their adolescent or young adult?
9. Do you permit parents to call for appointments, refills, update information, pain management advice?
  - No
  - Yes
10. Do you find that newly referred patients and their parents are prepared for treatment for the adult health care system?
  - No
  - If no, what do you believe is missing from their preparation? (open-ended question, free text).
  - Yes
11. Does your clinic provide support to newly transitioned young adult patients after hours?
  - No
  - Yes
  - If yes, who can the patient contact?
    - Physician
    - Registered nurse
    - Psychologist
    - Physiotherapist
    - Other: (free text)
12. What method is available for them to contact you after hours: (drop down and click whatever answer applies): phone, in person, email, pager, text
13. Does your clinic offer group sessions to people living with chronic pain?
  - No
  - Yes
  - If yes, do you offer sessions for young adults only?
    - No
    - If no, what is the average age of the group in these sessions?
    - Yes
    - If yes, what is the age range of participants for these young adult group sessions?
14. Do you provide support in advocating for school or university accommodations for younger patients?
  - No
  - Yes
  - If yes, how do you formally requested accommodations
    - Phone
    - Letter
    - Face-to-face meeting
    - Electronically (email)
15. What is the youngest age of a patient you have cared for in your clinic? (numerical age of patient)
16. Is there anything we did not ask about the care you provide for recently transitioned young people with chronic pain at your clinic that you feel we should know? (open-ended question, free text)
